# Toward an Expanded Focus for Occupational Safety and Health: A Commentary

**DOI:** 10.3390/ijerph16244946

**Published:** 2019-12-06

**Authors:** Paul A. Schulte, George Delclos, Sarah A. Felknor, L. Casey Chosewood

**Affiliations:** 1 National Institute for Occupational Safety and Health, Cincinnati, OH 45226, USA; SFelknor@cdc.gov (S.A.F.); ahx6@cdc.gov (L.C.C.); 2 Southwest Center for Occupational and Environmental Health, University of Texas Health Science Center School of Public Health, Houston, TX 77030, USA; George.Delclos@uth.tmc.edu

**Keywords:** well-being, systems thinking, future of work

## Abstract

Powerful and ongoing changes in how people work, the workforce, and the workplace require a more holistic view of each of these. We argue that an expanded focus for occupational safety and health (OSH) is necessary to prepare for and respond rapidly to future changes in the world of work that will certainly challenge traditional OSH systems. The WHO Model for Action, various European efforts at well-being, and the Total Worker Health concept provide a foundation for addressing changes in the world of work. However, a paradigm expansion to include the recognition of worker and workforce well-being as an important outcome of OSH will be needed. It will also be vital to stimulate transdisciplinary efforts and find innovative ways to attract and train students into OSH professions as the paradigm expands. This will require active marketing of the OSH field as vibrant career choice, as a profession filled with meaningful, engaging responsibilities, and as a well-placed investment for industry and society. An expanded paradigm will result in the need for new disciplines and specialties in OSH, which may be useful in new market efforts to attract new professionals. Ultimately, to achieve worker and workforce well-being we must consider how to implement this expanded focus.

## 1. Introduction

The changing nature of work, the workforce, and the workplace is widely recognized and discussed [1,2,3,4,5,6,7,8,9]. These changes, resulting from technology, globalization, shifts in demographics, and other economic and political forces, pose many potential problems for workers, employers, and society today and for the foreseeable future. With the goals of increasing productivity and greater incorporation of technology, the pace of work has intensified. Terms of employment have changed for many with nonstandard work (short-term contracts, gigs, platform work, etc.) more common. While these arrangements may represent more flexibility for employers, they can translate into more precarious employment for workers; lower pay for equivalent education, skills, and experience than those with long-term employment arrangements; fewer benefits; and greater turnover [5,10,11,12]. These outcomes can negatively affect the health of the workforce and their overall well-being. As a consequence, the focus of the occupational safety and health (OSH) field will need to be transformed to meet the needs of the future. This paper describes an approach to that transformation.

Factors influencing worker health and well-being go beyond traditional occupational safety and health (OSH) concerns (e.g., exposures to chemical, physical, or biological agents). They include changing demographic profiles (e.g., more women, immigrant, and older workers, more chronic disease and mental health conditions), varying employment arrangements and intensification of work organization demands, increasing psychosocial hazards, and changes in the built and natural environments [2,13,14,15,16,17,18,19]. These work-related factors combine with individual health and lifestyle and factors in the home, community, and general society to affect worker health and well-being. Considering work and nonwork influences is consistent with the holistic World Health Organization (WHO) global model for action, various European efforts for well-being, and the U.S. National Institute for Occupational Safety and Health (NIOSH) *Total Worker Health* ^®^ perspective which advocates policies, practices, and programs that both protect workers and prevent injury and disease, on and off the job [4,18,20,21,22].

To date, despite these notable pioneering efforts, little work has been done to examine this complex interaction between work and nonwork influences. However, further orienting the OSH field to more broadly focus on worker and workforce well-being presents a new way to do so, while addressing current and future changes [18]. The concept of worker well-being emphasizes quality of life, driven by the relationship between individual worker safety and health and factors both at and outside the workplace, and a desire for workers to thrive and achieve their full potential [16]. Well-being integrates, but goes beyond, the traditional OSH goal of protecting workers from occupational hazards, to include preventing illness and promoting worker health [4,15,16,23,24,25]. Although the concept preserves the need to continue addressing deadly hazards and risks that occur in many subsectors where traditional OSH is still necessary, it also has the potential to broaden OSH practice. Such an integrated approach changes the field of OSH and the preparation of its professionals. The concept recognizes the foundation that the *Total Worker Health* and various other European and international efforts have developed, builds upon that research and explores areas for further opportunity and action [4,18,20,21,22].

Focusing on worker well-being as an outcome requires a clarification of the paradigm beyond the prevention of workplace injury and illness or solely using the workplace as a venue for health promotion. The clarification calls for collaborative organizational leadership, proactive company policies, accountability, training, better engagement of management and employees, following benchmarks over time, and identifying opportunities for course correction, with continuous feedback when necessary [1,3,18,25,26]. Embracing this paradigm clarification requires a more expansive, systems-thinking approach to better integrate traditional OSH and personal and socioeconomic risk factors, both horizontally (broadening the range of factors that impact health) and vertically (from a short-term perspective to a work-life-continuum perspective).

This paradigm expansion will change how we conduct OSH research, train the future OSH workforce, and design forward-thinking policies and practices within organizations to maximize worker health, safety, and well-being [26]. However, creating this paradigm expansion is not meant to displace the current members of the OSH community but rather to build a platform on which OSH specialists can work more closely with non-OSH or new OSH specialists, doing so will leverage additional success and support for their work. The audience for this report is the OSH professional community, as well as communities in other disciplines interested in work, the workforce, and the workplace. The central issue is the rationale for training OSH professionals of the future.

## 2. Changes in the World of Work and Worker Well-Being

Historically, the OSH field addressed direct links between workplace hazards and worker mortality, morbidity, and injury. Basically, the goal was to have workers return home at the end of their shifts as healthy as they had arrived that day. This effort has been somewhat successful in reducing burden on workers and employers in developed countries [27]. However, whereas many of the classic hazards of work remain in certain industries (e.g., construction) and require continued attention [25,28], it is also true that the 21st century has introduced greater complexity in the nature of work and employment, workforce demographics, and the workplace [29]. The norm for work is no longer a career in one company; rather, it is many jobs and possibly many careers. The employment relationship is increasingly fragmented, as demonstrated by brief, temporary contracts, or other nonstandard work arrangements [2,17,30]. As workers have many different jobs and work arrangements, they may be exposed to multiple and simultaneous risk scenarios, and have increased periods of unemployment or underemployment [1,2,3,11,17]. These conditions can adversely affect the morbidity and mortality of workers over the working lifespan [31,32,33,34,35]. In some sectors, e.g., construction, few jobs are under the standard work arrangement typically found in manufacturing, healthcare, or other sectors.

Work and the workforce of the future will be intensely affected by technology, globalization, urbanization, sustainability pressures, and climate change [19,36,37,38,39]. It is quite likely that technology will extensively displace workers in the future [39]. Although historically technology has always changed the way work was done, the number of jobs ultimately created surpassed the number of jobs eliminated [40]. It is unclear if the Fourth Industrial Revolution (also known as Work 4.0) [41] will simply replace workers with technology or if some new equilibrium will be found with new job tasks ultimately created to support new technologies. In the process, many workers will likely continue to suffer from lost wages and the psychosocial and physical health effects of precarious employment, unemployment, and underemployment; they might also be unintentionally harmed by new hazards created by untested interventions [5,33,37,42,43,44,45,46].

**Figure 1:**
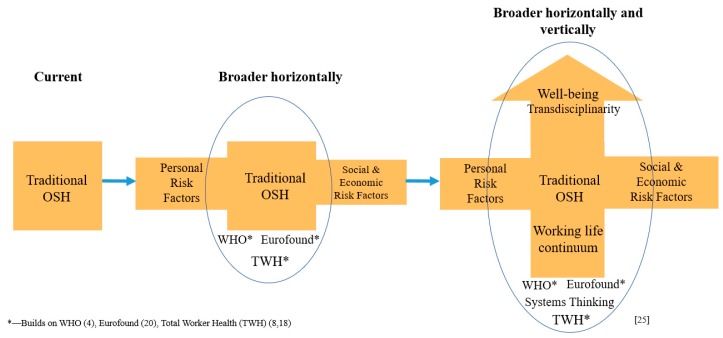
An expanded focus for occupational safety and health (OSH).

The demographic profile of the workforce from developed economies is shifting, often characterized by aging workers, a greater burden of chronic disease, and more women, and immigrants [3,5,9]. The workplace is likewise changing. A larger proportion of businesses are now small to medium size, but some sectors have increasing market concentration and are evolving into larger and fewer companies. More workers are telecommuting, and work plans and conditions have diversified [4]. In combination with individual health and lifestyle changes and factors in the home, community, and general society, these changes introduce new work-related risks that may disproportionately affect worker health and productivity. Such changes challenge traditional approaches to ensuring a safe and healthy workforce and worker well-being [38,44,45,46,47,48,49,50]. Consequently, there is a need to think of OSH in an expanded way, broader both horizontally and vertically (Figure 1) [47,48,49,50,51,52,53].

A broader horizontal view recognizes that the health of a worker and of the workforce is affected by not only occupational risk factors but also personal, family, and community-level risk factors; and these factors influence each other while impacting health outcomes [4,18,20,21,22,52]. This is consistent with the WHO Model for Action [4], various European initiatives such as in Finland and by Eurofound [20,21,22], and the *Total Worker Health* concept [8,18]. Addressing occupational risk factors without the others is incomplete, and vice versa, and may lead to bias and interventions that fail or are of marginal value. Hence, we must better define and evaluate this expanded view of worker well-being. A vertically broader focus looks along the entire working-life continuum, from pre-work to post-work [25,26,48]. Not only the health and productivity of the work and workforce are important; so is the health of the business enterprise. The latter is affected by—and also affects—the health of the workforce [38]; both deserve consideration.

### 2.1. Horizontal Thinking

A growing literature describes the interaction of occupational and nonoccupational risk factors. The history of this research goes back to at least the 1984 efforts of the World Health Organization (WHO) to promote consideration of the work-relatedness of disease [47]. This thinking further evolved in the early 2000s with the NIOSH Steps to a Healthier U.S. Workforce effort, followed by the Total Worker Health (TWH) program in 2011, [8] the WHO Global Action Model in 2010 [4] and an array of European efforts in worker well-being [20,21,22,53]. A broad list of factors pertinent to TWH have been identified (https://www.cdc.gov/niosh/twh/pdfs/twh-issues-4x3_10282015_final.pdf).

Underpinning these initiatives, 32 models of the interactions between occupation and personal risk factors were described in 2012 [52] for eight personal risk factors: genetics, age, gender, chronic disease, obesity, smoking, alcohol use, and prescription drug use. Subsequently, the interactions were studied in various in-depth analyses [54,55]. These interactions occur and “play out” within an economic and social environment that can strongly influence them [56,57,58,59,60,61,62]. Horizontal thinking is also illustrated by the WHO Healthy Workplace initiative [4]. Occupational risk factors include not only exposure to identified hazards but also refer to the organization, pacing, and intensity of work, the degree of demands, control and support, and the nature of relationships and social justice in an organization [46,60].

### 2.2. Vertical Thinking

**Figure 2:**
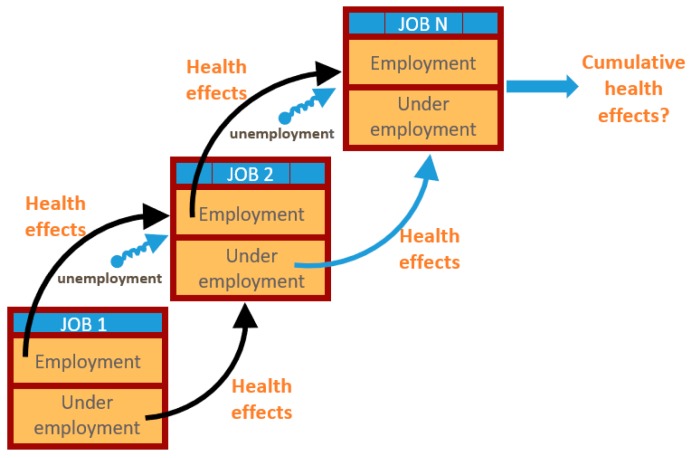
Health burden within and between jobs.

An expanded focus for OSH also needs to be longitudinal over the working life, that is, vertical. OSH should address hazards not only in a single job but also along the whole working-life continuum (Figure 2) [25,26,62,63]. This means addressing the precarious nature of work, its attendant stresses and income insecurities as well as the times and transitions between jobs, where unemployment and underemployment and the quality of work design can cause significant health problems [5,33,63,64,65]. Such conditions of employment are also occupational health hazards. Their incorporation into the traditional OSH framework, especially as strong influencers on worker well-being, will require substantial changes. The necessary changes involve how we conduct OSH research, train future OSH professionals, develop risk assessment and management strategies, and design interventions to maximize worker health and well-being [25,26]

Thinking about the working-life continuum implies addressing the cumulative nature of risks. Cumulative risk assessment and exposomic science are growing fields that acknowledge the accretion of risks over time [66,67,68]. This includes the accumulation of all types of risks: chemical, physical, biological, and psychosocial [68]. How this assessment will be used in the changing OSH environment is a critical, unanswered question.

## 3. Operationalizing the Concept of Worker Well-Being

There have been various efforts for operationalizing the concept of well-being [14,16,21,53,69,70]. Eurofound has included well-being in its surveys for many years and the European Quality of Life Survey assesses subjective well-being, access to care, aspects of individual quality of life including work-life balance, and care responsibility [22]. Allin [69] assessed how the UK measured well-being and how well-being could be added to the policy appraisal cycle. Another example is the TWH concept which illustrates a multifaceted framework for considering worker well-being. The rationale for TWH is that “systematic changes in the economy and sociodemographic workforce factors are rendering past approaches to protecting workers ineffective. Increasingly, timeline, and global competition…scientific evidence…now supports that risk factors in the workplace can contribute to common health problems previously considered unrelated to work” [70]. The focus of TWH is to address worker well-being through the integration of occupational safety and health protection with workplace policies, programs, and practices to prevent injury and illness and advance overall health and well-being [70]. Well-designed work not only safeguards workers but also can add meaning, purpose, fulfillment, and increased knowledge—all antecedents of greater life satisfaction, improved health opportunities, and healthier decision-making. This focus on well-being also reflects a shift in traditional OSH thinking, moving the goal posts toward having workers return home at the end of their shifts *healthier* than they had arrived that day. New standardized metrics to measure and operationalize the concept of work well-being are needed, since how people work and the work they do change quickly. NIOSH initiated efforts in 2016 to better define well-being in the context of work and recently summarized a several-year effort for expanding the OSH paradigm into a new framework for worker well-being [8,16,70]. This framework seeks to improve well-being measurement and provide tools for stakeholders to improve worker health and quality of life.

The framework, developed by a combination of systematic and targeted literature reviews, followed by expert input, resulted in five major domains: (1) workplace physical environment and safety climate; (2) workplace policies and culture; (3) health status; (4) work evaluations and experience; and (5) home, community, and society. These five domains, in turn, are subdivided into 20 subdomains and 58 subdomain constructs that provide progressive levels of detail on the framework. Two main methodological considerations drive composition of the framework, requiring that it incorporates (1) concepts and measures that better reflect workers’ lives, both within and outside the workplace, and (2) both subjective and objective approaches to better understand well-being. Whereas subjective measures characterize how individuals perceive their own quality of life, objective approaches can define a set of basic needs integral to achieving quality of life. Subjective and objective approaches may not always correlate (for example, a millionaire may be unsatisfied despite having all basic needs met or vice versa). Thus, neither subjective nor objective measures alone suffice; both have limitations, and both should be jointly considered [16,26].

## 4. Traditional and New OSH Skill Sets

Historically, OSH has been built on occupational medicine and nursing, industrial hygiene, safety epidemiology, toxicology, engineering, occupational psychology, and law. However, to address the changes in work, the workforce, and the workplace, we need a systems-based, more interprofessional approach to identify new skills that support expanded horizontal and vertical integration. These skills would include areas such as applied economics, sociology, anthropology, human relations, political science, gerontology, informatics, education, program evaluation, business, corporate social responsibility, climate science, architecture, urban planning, and sustainability. Clearly, aspects of some of these disciplines already contribute to OSH, but these contributions must grow, be disseminated, and be consistently operationalized; this is challenging.

Although important progress has occurred in worker well-being research, interventions, and partnerships, the development of training approaches for OSH professionals of the future to incorporate skills needed under the expanded paradigm is lagging behind.

The concept and practice of TWH should move beyond a centrally focused area of interest to one directed and enriched by partners and stakeholders from many sectors. To gain broader interest, uptake, and pertinence, the focus should shift to training OSH professionals to embrace this approach. In the U.S., the existing network of OSH academic training programs, with their foundation in traditional OSH, are central to this expanded training. Their access to students and local workplaces and their understanding of regional and emerging challenges in OSH, combined with their long history of multidisciplinary training in core and allied OSH programs, position them ideally to help expand reach. However, it is also clear that growth and expansion of the TWH program and other similar programs in Europe and elsewhere and their strategies may require the participation of a broader set of partners, stakeholders, and practitioners. Academic partners can develop and incorporate innovative curricula, teaching methods, and practice in collaboration with practitioners, employers, and unions, to make this next leap forward. An important challenge, however, will be how to provide acquisition of new skill sets, knowledge areas, interprofessionalism, and application of systems-thinking to OSH issues and challenges without overburdening the curriculum [26,71,72,73]. The dilemma is that there may be two different directions the OSH field could go. One is that future OSH experts could be taught to recognize when and how to collaborate with specialists in other fields (e.g., social workers and adult health promotion experts) toward achieving a common goal—worker well-being. That is, how to best engage a diverse set of stakeholders, typically in multiple sectors, coordinating a set of differentiated activities or interventions through a mutually reinforcing plan of action. The other direction would be one in which OSH specialists of the future are trained to know and cover all of these disciplines themselves, one where they possess skills to recognize the contribution of work and nonwork risks, and how to intervene proactively. This is a question for further resolution.

The timing for a paradigm expansion supported by innovative training methods in OSH is right. The European Union has included systems thinking among the core competencies for the public health professional [72]. Schools of public health in the U.S., led by the Council on Education for Public Health, are also reorienting public health education, emphasizing both systems-thinking (an approach that examines how things are interconnected, within a whole entity) and interprofessionalism (working with professionals outside the disciplines of public health) [73]. Additionally, there is increasing attention on Public Health 3.0 as a modernized approach to public health, aimed at improving health outcomes and extending life expectancy by focusing on social determinants, with work clearly an important determinant [74].

## 5. Efforts in Developing an Expanded Focus for OSH

Expanding earlier paradigms to adjust to changing conditions or work is not new. Early OSH professionals in the 19th century played an inspection and monitoring role to ensure compliance with existing laws, reporting to a central government or helping companies meet regulatory requirements. The late 19th century saw the development of both the generalist and specialist roles in OSH, which led to an expansion of the field with the professionalization and specialization of OSH disciplines. The last century saw an expanded focus for OSH in response to advances in technology and in hazard recognition and control, as well as an expanded understanding of the psychosocial and human factor influences on injury prevention [75].

Worker well-being, with notable precursors, was introduced in the early 21st century, expanding the traditional OSH role of preventing workplace risks and hazards to a broader role of promoting worker health and well-being as an objective of OSH programs [14,16,53,76,77,78]. The NIOSH *Total Worker Health* program was introduced in 2011, and provided an expanded framework for OSH to integrate protection from work-related hazards with promotion of injury and illness prevention efforts [8,18]. As we enter the transformative Fourth Industrial Revolution—in which fast-paced change is wielding its impact on the nature of work, the workforce, and the workplace—debate continues over the need for OSH generalists and specialists [35]. A paradigm expansion is needed to approach the middle of the 21st century with a framework that encompasses the broad range of determinants of worker and workforce health and safety. This also represents an opportunity to redefine the role of OSH professionals and once again move the field forward.

Various efforts are underway to expand the focus of OSH. One such effort is a joint project by NIOSH and the University of Texas School of Public Health Southwest Center for Occupational and Environmental Health, a NIOSH Education and Research Center (https://projectreporter.nih.gov/project_info_description.cfm?aid=9837226&icde=47536290&ddparam=&ddvalue=&ddsub=&cr=1&csb=default&cs=ASC&pball). Over a 3-year period, they will collaborate on a process to consider how to conduct OSH research, train the future OSH professional workforce, and design forward-thinking policies to maximize worker health and well-being. This collaboration will contribute to the public discourse on the future focus of OSH in a major way, through a series of international conferences and dissemination activities. These will bring together a broad, international, and interprofessional audience that includes but goes beyond employers, workers, and the academic community, focusing on the three critical areas of research, training, and policy/application. The unifying theme for this collaborative effort is the paradigm expansion in focus for OSH.

Another effort is the biannual Well-being at Work (WAW) conferences. Since 2010, this European conference series has developed a foundation for thinking about well-being, sharing state-of-the-art knowledge and best practices, and incorporating a broad range of disciplines. The most recent conference, held in May 2019 in Paris, extends the series (https://www.inrs-waw2019.fr/). This conference, supported by the French National Research and Safety Institute for the Prevention of Occupational Accidents and Diseases (INRS) and Partnership for European Research in Occupational Safety and Health (PEROSH), broadened the focus on well-being. PEROSH is the European network for research in occupational health, focusing on “well-being at work” as a strategic component of OSH in Europe.

A third effort, based on the concept of workplace innovation, goes beyond the material social contract, where workers had some control of or remuneration for their labors, to focus more on the working life and the development of capabilities (skills) in smart, adaptive jobs. This approach has led to the establishment of the European Workplace Innovation Network (EUWIN). EUWIN is a learning network that promotes Europe-wide knowledge-sharing on workplace innovation, open to organizations, stakeholders, policymakers, and researchers [79]. One objective of the EUWIN effort is simultaneously improving well-being at work and organizational performance. EUWIN describes workplace innovations as designing “new and combined interventions in work organization, human resource management, labor relations, and supportive technologies.” The nature of the innovations is participatory and inclusive, embedding workplace practices grounded in continuing reflection, learning, and improvement in the way organizations manage their employees, organize work, and deploy technologies. Although EUWIN does not have the holistic perspective of other concepts of well-being at work, it does focus on the broad range of forces in the workplace that affect a worker.

A fourth effort promotes achievement of capacity-building goals of the TWH research agenda. Table 1 lists selected goals for building TWH capacity among OSH professionals, and various universities are taking significant steps toward increasing the numbers of professionals trained in TWH approaches (http://www.cdc.gov/niosh/twh/confform.html). Additionally, TWH is part of The NIOSH National Occupational Research Agenda cross-sector on Healthy Work Design and Well-being, which is aimed at gathering information and encouraging research to expand the focus of occupational safety and health.

### 5.1. Systems Approach to OSH

Issues and problems that arise in OSH are often multifaceted and complex. Developing solutions to these issues involves analyzing and synthesizing data and information in a way that accounts for numerous intersecting factors, at different levels, that led to current work-related problems [43,57,58,59,60,61]. Using a traditional linear or fishbone approach to this analysis can be limited by not considering these various factors, stakeholders, and levels. Thus, an expanded focus for OSH requires systems-thinking [71,80,81,82,83].

Systems-thinking is a multifaceted cross-disciplinary effort with the following perspectives, as described by [81]: (1) increased attention to how new knowledge is gained, managed, exchanged, interpreted, integrated, and disseminated; (2) emphasis on a network-centric approach that encourages relationship-building among and between individuals and organizations across traditional disciplines and fields in order to achieve relevant goals and objectives; (3) development of models and projections, using a variety of analytic approaches (e.g., differential equations, agent-based modeling, and system-dynamic modeling) in order to improve strategic decision-making; and (4) systems organizing in order to foster improvements in organizational structures and functions [81,82].

The future hazards, exposures, risks, and interventions for workers, work, and the workplace cannot be viewed as separate occurrences or unaffected by macro trends or factors outside the workplace. A systems-centric approach should drive OSH research practice, policy development, and education. The transdisciplinary approach may underpin a systems-centric view for OSH. Key to such an approach is an integrative process that synthesizes and extends disciplinary-specific theories, concepts, or methods (or all three) to create new models and language to address OSH issues [84]. Systems-thinking in OSH will require addressing client-specific issues (e.g., confidential business information, worker right-to-know), and ownership of the fruits of production while also focusing on the evolving concepts of the nature of work, technological unemployment and underemployment, and the bidirectional relationships between occupational and non-occupational risk factors. Systems thinking will also require new ways to manage systems knowledge, new networks and methods for analyzing complex systems, and new adaptive systems [81]. OSH professionals will need to approach these requirements with skill sets appropriate to these tasks.

### 5.2. Future of the OSH Professions

Efforts to expand the focus of OSH are occurring simultaneously with exponential growth in information technology, making information much more accessible to employers, workers, and various decision-makers. This may give them the feeling of having the expertise formerly provided by professionals in the OSH field. The future of the professions in general, and OSH in particular, merits consideration because technology may transform the work of experts and specialists [85]. There are already examples of technologically induced displacement involving radiologists, lawyers, and architects [86]. Technology can be a powerful enabling factor but can also be disruptive. The challenge for OSH and other professions will be to use technologies (e.g., artificial intelligence, digitalization, robotics, sensors, and automation) to help develop and provide expertise within and across relevant disciplines.

For all information seekers, increasingly practical expertise will be available online. Future demand for professional education may be for more micro-credit training rather than multi-year graduate training. Still, there will be a major role for professionals as generators of new information, interpreters of information, and advisors on risk management decisions. In expanding the focus of OSH, practitioners will learn from both online information and formal education and training. Each of the OSH professions will need to evolve in regard to technological influences and other macro trends [87]. However, this will benefit from a more systematic and formal approach.

Three approaches to expand OSH training can be envisioned. One pertains to increasing the knowledge base and skill sets of OSH practitioners and investigators with augmented training and systems-thinking [81,88]. The second is to engage other professions to expand the focus of OSH and develop collaborations and partnerships [89]. The third would be a combination of both. For all three approaches to focus on well-being as an outcome of OSH activity, a holistic analysis of worker health and well-being is needed.

Collaborative approaches among professions have been variously defined, but the definitions of multidisciplinary, interdisciplinary, and transdisciplinary described by Rosenfeld [90] may serve as benchmarks:

Multidisciplinary: researchers and practitioners work in parallel or sequentially from a disciplinary-specific base to address common problems.

Interdisciplinary: researchers and practitioners work jointly but still from a disciplinary-specific basis to address common problems.

Transdisciplinary: researchers and practitioners work jointly, using a shared conceptual framework drawing together disciplinary-specific theories, concepts, and approaches to address common problems.

Addressing future OSH issues will require an integrative model, the kind prototypically illustrated by the work of Sorensen et al. [91] and presented by many others [52,92,93,94]. An expanded focus for OSH also may continue to use, and more widely promote, a transdisciplinary approach. Transdisciplinary research has been defined as a “process in which team members representing different fields work together over extended periods to develop shared conceptual and methodological frameworks that not only integrate but transcend their disciplinary perspectives” [84]. What is missing is how the organizational and institutional context/environment in which OSH professionals are trained and operate can support transdisciplinary collaboration between them and their holistic approaches with employers. Additionally, important is knowledge of organizational and institutional barriers and how they can be overcome.

The issue of “who owns what” in the OSH field of the 19th and 20th centuries is again pertinent, and at a time in which a holistic focus is advocated, there are likely to be questions and conflicts among professions. As Hale [75] has observed:

“Given the tendency of aspiring professions to stake a claim to ownership of specific tasks, models, and methods, which should be permitted to own what? Was the knowledge of the full range of hazards and their prevention across all industries so broad that it was beyond the capability of one specialist to understand and advise on it? If so, how could that breadth of knowledge be best divided between those competing professions to ensure a viable depth in each area coupled with effective communication and collaboration across the divides between them?”

Developing the expanded focus for OSH will thus require a collaborative approach to working arrangements among different professions, new transdisciplinary curricula, enhanced research on burden of work and the determinants of well-being. Ultimately, it may require new public and corporate policies [14,15].

### 5.3. Active Marketing of OSH

The OSH community is being threatened and needs to defend itself and its relevance more than in past decades [95,96,97]. Governments, corporations, and universities are underfunding, downsizing, transferring, or eliminating the OSH concentration or focus. In part, this is because the actual and extensive burden of work-related injury, illness, and distress, in human and economic terms, may not be adequately portrayed or communicated [48,50,56,98,99].

All countries, particularly developed countries, have a great need for the OSH profession to address three macro trends: demographic transitions affecting workforces (e.g., the aging worker demographic); accelerating technological disruption; and the impact of globalization on the economy. The most compelling reason that the OSH profession is best suited to address these issues is they all involve work and workforce, the principal focus of the field. Regarding the aging workforce, productive aging strategies can be implemented directly from current work [100]. For technological disruption, the OSH field is poised to address work-related stress and work organization issues, but it needs to focus more on technological unemployment, underemployment, and skill gap issues. This will be difficult as current trends promote capital investments over labor [40], driven in part by the rapidly expanding growth of technology. The third focal area pertains to globalization and its impact on the economy. Clearly, certain global forces are diminishing the well-being of workers in both developed and developing countries: from “rust belt” plant closings to large, risks of occupational injury and illness in recurring waves of immigrants and migrant workers [12,101,102].

There is value in viewing OSH in a new light, positioning it in the mainstream of dealing with critical national and international problems [77]. The well-being of the workforce is directly tied to the productivity of the enterprise and to the well-being of a nation, but the OSH field has not made that case strongly. There is a need to actively market OSH to policymakers, prospective students, and the general public by extensively communicating the widespread burden of worker injury, illness, death, distress, and lack of individual well-being, and their strong impact on national productivity, competitiveness, and population well-being [24,77,103]. Such marketing is needed to further expand, enrich, and promote the field and the opportunity for it to address critical national and international problems.

The OSH field also needs to actively market to those who would consider careers in it. As has been previously observed [77]:

“Additionally, the field is suffering from a diminishing workforce and academic base due, in part, to the lack of good quality data on the magnitude and burden of work-related disease and disability, which does not argue that this is an area for career investment…. An image and perception problem exists. Young people considering entering a health field do not see OSH as highly relevant to the problems of the day. If the OSH field is going to accept the challenge to step into the breach of the coming demographic collision, it needs to reconfigure, reinvest in, and re-energize itself.”

## 6. Concerns and Issues about an Expanded Focus of OSH

There is concern that a paradigm expansion to well-being as an outcome, and more of a “public health approach” than a “labor approach,” could have negative implications. It is key to acknowledge that OSH has limited resources, not nearly enough to address problems under the current paradigm [104]. However, if the burden and magnitude of adverse OSH outcomes and the benefits of healthy work and well-being are expansively described, then the opportunity for more resources may increase. More to the point, it is no longer effective to think of OSH issues separate from the larger sphere of public health. Such an approach also means that, as Harrison and Dawson [24] note:

“OH [occupational health] practitioners will be concerned with not only employed workers, that is, those under some form of employment contract, but also self-employed and informal workers. Interventions will extend to families and communities, and will not be restricted to actions at the workplace. The promotion and maintenance of health and well-being will involve a consideration of all health determinants and will not be restricted to work-related health issues.

Importantly, the health of workers will not be seen as only the responsibility of employers, but also of the wider stakeholder group, including health, work, and environment authorities; insurance companies; and other healthcare practitioners. An example might be in addressing stress at work.”

Other potential negative implications include:

1. Employer/managers may emphasize individual approaches such as vitality/fitness and coping behavior, instead of primary stress prevention such as job autonomy, skill discretion, collaborative organizational leadership, or reducing physical workload.
2. Employers/managers may reject responsibility because the health problems are related to stress in private life, sports, etc.
3. Given all the technologies to monitor their workers, employers/managers might focus on selecting only healthy workers for certain jobs, instead of helping all workers improve their health.
4. Employers/managers may reduce “employability” to vitality/fitness instead of creating a learning work environment (Reviewer’s comment. This is a personal communication in a review of the paper).

Finally, the change in focus might lead to “blaming the worker” for adverse outcomes in the work environment. The expanded paradigm must stipulate that consideration of occupational and personal risk factors is predicated on “the primacy of traditional health protection, which prioritizes employer responsibilities for the organization of work over individual health behaviors” [105]. However, “if a higher- level conceptualization of well-being is pursued, which subsumes health, is aspirational, and includes reaching human potential…,” then various parties in society (governments, insurance companies, unions, and nongovernmental organizations, for instance) as well as workers will have responsibilities in promoting well-being [15].

The focus on well-being of the workforce and the workplace raises uncertainties and concerns for employers, who fear being “asked” to take on responsibilities that go beyond traditional OSH responsibilities. There is a need to listen to their concerns but use this as an approach to concurrently train them in their own paradigm expansion toward worker well-being. Already, there are many good examples of larger companies pioneering or adopting “expanded focus” types of approaches [14,106].

Additionally, there may be pushback by various agencies that focus on preventing specific diseases, or resistance from powerful forces in society that support traditional OSH. There will need to be efforts made to include these groups going forward and work collaboratively with them to address their concerns.

## 7. Conclusions

The OSH field (and component professions) must expand its focus to address the many changes in the nature of work, the workforce, and the workplace. Factors inside and outside work interact and influence the health of workers and the productivity of enterprises. We need to position ourselves to address both current and future changes and challenges. An expanded focus should include a broader view of the traditional workplace risk assessment that considers viewing and addressing interactions of work and nonwork factors, as well as the changes that arise over the working life. The concept of well-being represents a holistic, inclusive outcome that can be used to encompass the impact of this broad range of changes. However, well-being needs to be more actively operationalized, and interventions to achieve it need to be developed and tested.

To address this flux in work, workers, and places, the OSH field will need to develop a broader vision, develop and use new skill sets, and partner with other disciplines and new stakeholders. OSH professionals must learn to work transdisciplinarily. The field of OSH must place itself in the mainstream discourse around well-being of nations, competitiveness, and productivity by showing that it can contribute to these outcomes. For all this to happen, OSH must be a growing, vibrant discipline. Expanding the vision and growing the field of OSH could produce a healthier workforce and enhance the well-being of nations.

## Figures and Tables

**Table 1:** National agenda to develop Total Worker Health (TWH) capacity among occupational safety and health professions ^1^.

Activity/Output Goals
4.1.1: Survey key stakeholders to identify TWH training needs for existing and future workplace and allied professionals (that is, occupational safety and health, worksite health promotion, human resources, business, academic, policy, and other occupational and health professionals).
4.1.2: Identify existing educational programs and curricula where TWH education is currently offered and where TWH principles can be incorporated.
4.1.3: Assess the need for creating specialized TWH degrees, certificates, and continuing education programs.
4.1.4: Develop standard TWH core competencies to be used across programs.
4.1.5: Develop guidance in consultation with educators and academic professional societies and organizations for incorporating TWH core competencies into the curricula of existing and new degree, certificate, and continuing education programs.
4.1.6: Offer TWH seminars, workshops, and courses for undergraduate and graduate students, across various disciplines, e.g., Occupational Health, Public Health, Health Promotion, Health Sciences, Psychology and other Social Sciences, Business, Human Relations, and Engineering.
4.1.7: Evaluate mechanisms by which federal, state, and local agencies can support and fund TWH educational activities.
4.1.8: Publish accessible works in theory, research, and practice on integrative prevention strategies for worker safety, health, and well-being to train both existing and new TWH professionals.
4.2.3: Create a TWH professional organization or align with an existing professional organization to develop standards accreditation, and evaluation guidelines for TWH professionals to enhance their development and build capacity.

^1^ These are selected from a broader list of goals [70].
